# Supplementary material for: Chimpanzee brain morphometry utilizing standardized MRI preprocessing and macroanatomical annotations
Source: eLife. 2020 Nov 23;9:e60136. doi: 10.7554/eLife.60136 (PMC7723405; doi:10.7554/eLife.60136)
Supplement: Figure 2—source data 1. [file elife-60136-fig2-data1.docx]

**Source file for Complete List of Davi130 Labels**

| **Label Number** | **Davi130 Label** | **Brain Region** | **Hemisphere** | **Acronyms** |
| --- | --- | --- | --- | --- |
| 1 | Anterior Superior Frontal Gyrus | Frontal | left | L.aSFG |
| 2 | Anterior Superior Frontal Gyrus | Frontal | right | R.aSFG |
| 3 | Middle Superior Frontal Gyrus | Frontal | left | L.mSFG |
| 4 | Middle Superior Frontal Gyrus | Frontal | right | R.mSFG |
| 5 | Posterior Superior Frontal Gyrus | Frontal | left | L.Hth |
| 6 | Posterior Superior Frontal Gyrus | Frontal | right | R.pSFG |
| 7 | Anterior Middle Frontal Gyrus | Frontal | left | L.aMFG |
| 8 | Anterior Middle Frontal Gyrus | Frontal | right | R.aMFG |
| 9 | Posterior Middle Frontal Gyrus | Frontal | left | L.pMFG |
| 10 | Posterior Middle Frontal Gyrus | Frontal | right | R.pMFG |
| 11 | Anterior Inferior Frontal Gyrus | Frontal | left | L.aIFG |
| 12 | Anterior Inferior Frontal Gyrus | Frontal | right | R.aIFG |
| 13 | Middle Inferior Frontal Gyrus | Frontal | left | L.mIFG |
| 14 | Middle Inferior Frontal Gyrus | Frontal | right | R.mIFG |
| 15 | Posterior Inferior Frontal Gyrus | Frontal | left | L.pIFG |
| 16 | Posterior Inferior Frontal Gyrus | Frontal | right | R.pIFG |
| 17 | Medial Orbitofrontal Cortex | Frontal | left | L.mOFC |
| 18 | Medial Orbitofrontal Cortex | Frontal | right | R.mOFC |
| 19 | Lateral Orbitofrontal Cortex | Frontal | left | L.lOFC |
| 20 | Lateral Orbitofrontal Cortex | Frontal | right | R.lOFC |
| 21 | Anterior Cingulate Cortex | Limbic | left | L.ACC |
| 22 | Anterior Cingulate Cortex | Limbic | right | R.ACC |
| 23 | Middle Cingulate Cortex | Limbic | left | L.MCC |
| 24 | Middle Cingulate Cortex | Limbic | right | R.MCC |
| 25 | Posterior Cingulate Cortex | Limbic | left | L.PCC |
| 26 | Posterior Cingulate Cortex | Limbic | right | R.PCC |
| 27 | Superior Precentral Gyrus | Frontal | left | L.sPrCG |
| 28 | Superior Precentral Gyrus | Frontal | right | R.sPrCG |
| 29 | Middle Precentral Gyrus | Frontal | left | L.mPrCG |
| 30 | Middle Precentral Gyrus | Frontal | right | R.mPrCG |
| 31 | Inferior Precentral Gyrus | Frontal | left | L.iPrCG |
| 32 | Inferior Precentral Gyrus | Frontal | right | R.iPrCG |
| 33 | Paracentral Lobule | Parietal | left | L.PCL |
| 34 | Paracentral Lobule | Parietal | right | R.PCL |
| 35 | Frontal Operculum | Frontal | left | L.FOP |
| 36 | Frontal Operculum | Frontal | right | R.FOP |
| 37 | Parietal Operculum | Parietal | left | L.POP |
| 38 | Parietal Operculum | Parietal | right | R.POP |
| 39 | Anterior Insula | Temporal | left | L.aIns |
| 40 | Anterior Insula | Temporal | right | R.aIns |
| 41 | Posterior Insula | Temporal | left | L.pIns |
| 42 | Posterior Insula | Temporal | right | R.pIns |
| 43 | Anterior Transverse Temporal Gyrus | Temporal | left | L.aTTG |
| 44 | Anterior Transverse Temporal Gyrus | Temporal | right | R.aTTG |
| 45 | Posterior Transverse Temporal Gyrus | Temporal | left | L.pTTG |
| 46 | Posterior Transverse Temporal Gyrus | Temporal | right | R.pTTG |
| 47 | Anterior Superior Temporal Gyrus | Temporal | left | L.aSTG |
| 48 | Anterior Superior Temporal Gyrus | Temporal | right | R.aSTG |
| 49 | Posterior Superior Temporal Gyrus | Temporal | left | L.pSTG |
| 50 | Posterior Superior Temporal Gyrus | Temporal | right | R.pSTG |
| 51 | Anterior Middle Temporal Gyrus | Temporal | left | L.aMTG |
| 52 | Anterior Middle Temporal Gyrus | Temporal | right | R.aMTG |
| 53 | Posterior Middle Temporal Gyrus | Temporal | left | L.pMTG |
| 54 | Posterior Middle Temporal Gyrus | Temporal | right | R.pMTG |
| 55 | Anterior Inferior Temporal Gyrus | Temporal | left | L.aITG |
| 56 | Anterior Inferior Temporal Gyrus | Temporal | right | R.aITG |
| 57 | Posterior Inferior Temporal Gyrus | Temporal | left | L.pITG |
| 58 | Posterior Inferior Temporal Gyrus | Temporal | right | R.pITG |
| 59 | Entorhinal Cortex | Limbic | left | L.EHC |
| 60 | Entorhinal Cortex | Limbic | right | R.EHC |
| 61 | Anterior Fusiform Gyrus | Temporal | left | L.aFFG |
| 62 | Anterior Fusiform Gyrus | Temporal | right | R.aFFG |
| 63 | Posterior Fusiform Gyrus | Temporal | left | L.pFFG |
| 64 | Posterior Fusiform Gyrus | Temporal | right | R.pFFG |
| 65 | Parahippocampal Gyrus | Limbic | left | L.PHC |
| 66 | Parahippocampal Gyrus | Limbic | right | R.PHC |
| 67 | Amygdala | Limbic | left | L.Amy |
| 68 | Amygdala | Limbic | right | R.Amy |
| 69 | Hippocampus | Limbic | left | L.HC |
| 70 | Hippocampus | Limbic | right | R.HC |
| 71 | Superior Postcentral Gyrus | Parietal | left | L.sPoCG |
| 72 | Superior Postcentral Gyrus | Parietal | right | R.sPoCG |
| 73 | Middle Postcentral Gyrus | Parietal | left | L.mPoCG |
| 74 | Middle Postcentral Gyrus | Parietal | right | R.mPoCG |
| 75 | Inferior Postcentral Gyrus | Parietal | left | L.iPoCG |
| 76 | Inferior Postcentral Gyrus | Parietal | right | R.iPoCG |
| 77 | Superior Parietal Lobule | Parietal | left | L.SPL |
| 78 | Superior Parietal Lobule | Parietal | right | R.SPL |
| 79 | Supramarginal Gyrus | Parietal | left | L.SMG |
| 80 | Supramarginal Gyrus | Parietal | right | R.SMG |
| 81 | Angular Gyrus | Parietal | left | L.AnG |
| 82 | Angular Gyrus | Parietal | right | R.AnG |
| 83 | Precuneus | Parietal | left | L.PCun |
| 84 | Precuneus | Parietal | right | R.PCun |
| 85 | Cuneus | Occipital | left | L.Cun |
| 86 | Cuneus | Occipital | right | R.Cun |
| 87 | Lingual Gyrus | Occipital | left | L.LG |
| 88 | Lingual Gyrus | Occipital | right | R.LG |
| 89 | Calcarine Sulcus | Occipital | left | L.Calc |
| 90 | Calcarine Sulcus | Occipital | right | R.Calc |
| 91 | Superior Occipital Gyrus | Occipital | left | L.sOG |
| 92 | Superior Occipital Gyrus | Occipital | right | R.sOG |
| 93 | Middle Occipital Gyrus | Occipital | left | L.mOG |
| 94 | Middle Occipital Gyrus | Occipital | right | R.mOG |
| 95 | Inferior Occipital Gyrus | Occipital | left | L.iOG |
| 96 | Inferior Occipital Gyrus | Occipital | right | R.iOG |
| 97 | Caudate Nucleus | Basal Ganglia | left | L.CN |
| 98 | Caudate Nucleus | Basal Ganglia | right | R.CN |
| 99 | Nucleus Accumbens | Basal Ganglia | left | L.NA |
| 100 | Nucleus Accumbens | Basal Ganglia | right | R.NA |
| 101 | Basal Forebrain Nuclei | Basal Ganglia | left | L.BF |
| 102 | Basal Forebrain Nuclei | Basal Ganglia | right | R.BF |
| 103 | Putamen | Basal Ganglia | left | L.Pu |
| 104 | Putamen | Basal Ganglia | right | R.Pu |
| 105 | Globus pallidus | Basal Ganglia | left | L.GP |
| 106 | Globus pallidus | Basal Ganglia | right | R.GP |
| 107 | Thalamus | Basal Ganglia | left | L.Th |
| 108 | Thalamus | Basal Ganglia | right | R.Th |
| 109 | Hypothalamus | Basal Ganglia | left | L.HTh |
| 110 | Hypothalamus | Basal Ganglia | right | R.HTh |
| 111 | Cerebellum IX-Tonsil | Cerebellum | left | L.CerIX |
| 112 | Cerebellum IX-Tonsil | Cerebellum | right | R.CerIX |
| 113 | Cerebellum VIIIAB-Inferior Posterior - PML | Cerebellum | left | L.CerVIII |
| 114 | Cerebellum VIIIAB-Inferior Posterior - PML | Cerebellum | right | R.CerVIII |
| 115 | Cerebellum VIIA - Superior Posterior - Crus I of Ansiform Lobule | Cerebellum | left | L.CrusI |
| 116 | Cerebellum VIIA - Superior Posterior – Crus I of Ansiform Lobule | Cerebellum | right | R.CrusI |
| 117 | Cerebellum VIIA - Superior Posterior – Crus II of Ansiform Lobule with Paramedian 1 | Cerebellum | left | L.CrusII |
| 118 | Cerebellum VIIA-Superior Posterior - Crus II of Ansiform Lobule with Paramedian 1 | Cerebellum | right | R.CrusII |
| 119 | Cerebellum VI-Superior Posterior | Cerebellum | left | L.CerVI |
| 120 | Cerebellum VI-Superior Posterior | Cerebellum | right | R.CerVI |
| 121 | Cerebellum V-Anterior B | Cerebellum | left | L.CerVB |
| 122 | Cerebellum V-Anterior B | Cerebellum | right | R.CerVB |
| 123 | Cerebellum V-Anterior A | Cerebellum | left | L.CerVA |
| 124 | Cerebellum V-Anterior A | Cerebellum | right | R.CerVA |
| 125 | Cerebellum IV-Anterior Quadrangulate | Cerebellum | left | L.CerIV |
| 126 | Cerebellum IV-Anterior Quadrangulate | Cerebellum | right | R.CerIV |
| 127 | Cerebellum III-Anterior Quadrangulate | Cerebellum | left | L.CerIII |
| 128 | Cerebellum III-Anterior Quadrangulate | Cerebellum | right | R.CerIII |
| 129 | Cerebellum II-Anterior Quadrangulate | Cerebellum | left | L.CerII |
| 130 | Cerebellum II-Anterior Quadrangulate | Cerebellum | right | R.CerII |
