# Supplementary material for: Chimpanzee brain morphometry utilizing standardized MRI preprocessing and macroanatomical annotations
Source: eLife. 2020 Nov 23;9:e60136. doi: 10.7554/eLife.60136 (PMC7723405; doi:10.7554/eLife.60136)
Supplement: Figure 4—source data 1. [file elife-60136-fig4-data1.docx]

**Aging Effect on Gray Matter in Complete Davi130 Labels**

| **Davi130 Label** | **T-statistic** | **p-value** |
| --- | --- | --- |
| **Frontal Cortex** |  |  |
| Anterior Superior Frontal Gyrus (L.aSFG)* | -3.78 | 2.1x10^-4^ |
| Anterior Superior Frontal Gyrus (R.aSFG)* | -3.95 | 1.1x10^-4^ |
| Middle Superior Frontal Gyrus (L.mSFG)* | -4.75 | 4.1x10^-6^ |
| Middle Superior Frontal Gyrus (R.mSFG)* | -4.96 | 1.6x10^-6^ |
| Posterior Superior Frontal Gyrus (L.pSFG)* | -5.71 | 4.3x10^-8^ |
| Posterior Superior Frontal Gyrus (R.pSFG)* | -5.49 | 1.3x10^-7^ |
| Anterior Middle Frontal Gyrus (L.aMFG) | -2.80 | 0.0056 |
| Anterior Middle Frontal Gyrus (R.aMFG) | -3.12 | 0.0021 |
| Posterior Middle Frontal Gyrus (L.pMFG)* | -4.41 | 1.8x10^-5^ |
| Posterior Middle Frontal Gyrus (R.pMFG)* | -4.74 | 4.1x10^-6^ |
| Anterior Inferior Frontal Gyrus (L.aIFG) | -2.13 | 0.0341 |
| Anterior Inferior Frontal Gyrus (R.aIFG) | -2.68 | 0.0079 |
| Middle Inferior Frontal Gyrus (L.mIFG) | -3.35 | 0.0010 |
| Middle Inferior Frontal Gyrus (R.mIFG) | -3.73 | 0.0003 |
| Posterior Inferior Frontal Gyrus (L.pIFG)* | -4.55 | 9.7x10^-6^ |
| Posterior Inferior Frontal Gyrus (R.pIFG)* | -3.64 | 0.0004 |
| Medial Orbitofrontal Cortex (L.mOFC) | -2.90 | 0.0041 |
| Medial Orbitofrontal Cortex (R.mOFC) | -3.22 | 0.0015 |
| Lateral Orbitofrontal Cortex (L.lOFC)* | -4.37 | 2.1x10^-5^ |
| Lateral Orbitofrontal Cortex (R.lOFC)* | -4.54 | 1.0x10^-5^ |
| Superior Precentral Gyrus (L.sPrCG)* | -4.31 | 2.6x10^-5^ |
| Superior Precentral Gyrus (R.sPrCG) | -3.42 | 0.0008 |
| Middle Precentral Gyrus (L.mPrCG)* | -4.93 | 1.8x10^-6^ |
| Middle Precentral Gyrus (R.mPrCG)* | -3.57 | 0.0005 |
| Inferior Precentral Gyrus (L.iPrCG)* | -4.11 | 5.92x10^-5^ |
| Inferior Precentral Gyrus (R.iPrCG)* | -3.53 | 0.0005 |
| Frontal Operculum (L.FOP) | -3.36 | 0.0009 |
| Frontal Operculum (R.FOP) | -3.17 | 0.0018 |
|  |  |  |
| **Limbic Cortex** |  |  |
| Anterior Cingulate Gyrus (L.ACC)* | -4.17 | 4.7x10^-5^ |
| Anterior Cingulate Gyrus (R.ACC)* | -3.74 | 0.0002 |
| Middle Cingulate Gyrus (L.MCC)* | -4.52 | 1.1x10^-5^ |
| Middle Cingulate Gyrus (R.MCC)* | -3.61 | 0.0004 |
| Posterior Cingulate Gyrus (L.PCC)* | -4.17 | 4.6x10^-5^ |
| Posterior Cingulate Gyrus (R.PCC)* | -3.54 | 0.0005 |
| Entorhinal Cortex (L.EnC) | -0.40 | 0.6903 |
| Entorhinal Cortex (R.EnC) | -2.10 | 0.0368 |
| Parahippocampal Gyrus (L.PHC) | -2.57 | 0.0110 |
| Parahippocampal Gyrus (R.PHC) | -3.41 | 0.0008 |
| Amygdala (L.Amy) | -3.31 | 0.0011 |
| Amygdala (R.Amy) | -3.01 | 0.0030 |
| Hippocampus (L.HC) | -1.02 | 0.3100 |
| Hippocampus (R.HC) | -2.56 | 0.0114 |
|  |  |  |
| **Temporal Cortex** |  |  |
| Anterior Insula (L.aIns) | -3.26 | 0.0013 |
| Anterior Insula (R.aIns)* | -3.80 | 0.0002 |
| Posterior Insula (L.pIns) | -2.84 | 0.0050 |
| Posterior Insula (R.pIns) | -3.00 | 0.0031 |
| Anterior Transverse Temporal Gyrus (L.aTTG* | -3.89 | 0.0001 |
| Anterior Transverse Temporal Gyrus (R.aTTG) | -3.33 | 0.0010 |
| Posterior Transverse Temporal Gyrus (R.pTTG)* | -3.47 | 0.0006 |
| Posterior Transverse Temporal Gyrus (L.pTTG) | -3.16 | 0.0018 |
| Anterior Superior Temporal Gyrus (L.aSTG) | -3.39 | 0.0009 |
| Anterior Superior Temporal Gyrus (R.aSTG) | -3.11 | 0.0021 |
| Posterior Superior Temporal Gyrus (L.pSTG)* | -4.86 | 2.5x10^-6^ |
| Posterior Superior Temporal Gyrus (R.pSTG)* | -5.40 | 2.0x10^-7^ |
| Anterior Middle Temporal Gyrus (L.aMTG)* | -3.80 | 0.0002 |
| Anterior Middle Temporal Gyrus (R.aMTG)* | -3.55 | 0.0005 |
| Posterior Middle Temporal Gyrus (L.pMTG) | -2.60 | 0.0101 |
| Posterior Middle Temporal Gyrus (R.pMTG) | -3.19 | 0.0017 |
| Anterior Inferior Temporal Gyrus (L.aITG) | -2.43 | 0.0162 |
| Anterior Inferior Temporal Gyrus (R.aITG) | -2.71 | 0.0074 |
| Posterior Inferior Temporal Gyrus (L.pITG) | -3.12 | 0.0021 |
| Posterior Inferior Temporal Gyrus (R.pITG) | -3.20 | 0.0016 |
| Anterior Fusiform Gyrus (L.aFFG) | -3.07 | 0.0024 |
| Anterior Fusiform Gyrus (R.aFFG) | -3.20 | 0.0016 |
| Posterior Fusiform Gyrus (L.pFFG) | -3.42 | 0.0008 |
| Posterior Fusiform Gyrus (R.pFFG) | -3.05 | 0.0026 |
|  |  |  |
| **Parietal Cortex** |  |  |
| Superior Postcentral Gyrus (L.sPoCG) | -3.21 | 0.0015 |
| Superior Postcentral Gyrus (R.sPoCG) | -2.48 | 0.0140 |
| Middle Postcentral Gyrus (L.mPoCG) | -2.96 | 0.0035 |
| Middle Postcentral Gyrus (R.mPoCG) | -2.19 | 0.0294 |
| Inferior Postcentral Gyrus (L.iPoCG) | -2.80 | 0.0056 |
| Inferior Postcentral Gyrus (R.iPoCG) | -2.59 | 0.0103 |
| Superior Parietal Lobule (L.SPL) | -1.64 | 0.1027 |
| Superior Parietal Lobule (R.SPL) | -2.03 | 0.0435 |
| Supramarginal Gyrus (L.SMG) | -2.97 | 0.0033 |
| Supramarginal Gyrus (R.SMG) | -2.80 | 0.0056 |
| Angular Gyrus (L.AG) | -3.21 | 0.0015 |
| Angular Gyrus (R.AG) | -2.84 | 0.0050 |
| Parietal Operculum (L.POP) | -3.29 | 0.0012 |
| Parietal Operculum (R.POP) | -2.36 | 0.0193 |
| Paracentral Lobule (L.PCL)* | -4.11 | 5.8x10^-5^ |
| Paracentral Lobule (R.PCL) | -2.14 | 0.0332 |
| Precuneus (L.PCun)* | -3.87 | 0.0002 |
| Precuneus (R.PCun)* | -3.71 | 0.0003 |
|  |  |  |
| **Occipital** |  |  |
| Cuneus (L.Cun)* | -3.74 | 0.0002 |
| Cuneus (R.Cun) | -1.84 | 0.0668 |
| Lingual Gyrus (L.LG)* | -5.03 | 1.1x10^-6^ |
| Lingual Gyrus (R.LG)* | -4.84 | 2.7x10^-6^ |
| Calcarine Sulcus (R.Calc)* | -4.69 | 5.2x10^-6^ |
| Calcarine Sulcus (L.Calc)* | -4.46 | 1.4x10^-5^ |
| Superior Occipital Gyrus (L.sOG) | -1.66 | 0.0996 |
| Superior Occipital Gyrus (R.sOG) | -1.25 | 0.2112 |
| Middle Occipital Gyrus (L.mOG) | 0.05 | 0.9603 |
| Middle Occipital Gyrus (R.mOG) | 0.22 | 0.8275 |
| Inferior Occipital Gyrus (L.iOG) | -1.36 | 0.1747 |
| Inferior Occipital Gyrus (R.iOG) | -1.36 | 0.1759 |
|  |  |  |
| **Basal Ganglia** |  |  |
| Caudate Nuclues (L.CN)* | -4.21 | 3.9x10^-5^ |
| Caudate Nuclues (R.CN)* | -4.68 | 5.5x10^-6^ |
| Nucleus Accumbens (L.NA)* | -5.04 | 1.1x10^-6^ |
| Nucleus Accumbens (R.NA)* | -4.24 | 3.5x10^-5^ |
| Basal Forebrain Nuclei (L.BF) | -2.98 | 0.0033 |
| Basal Forebrain Nuclei (R.BF) | -2.77 | 0.0061 |
| Putamen (L.Pu)* | -4.41 | 1.7x10^-5^ |
| Putamen (R.Pu)* | -6.16 | 4.3x10^-9^ |
| Globus Pallidus (L.GP) | 1.88 | 0.0610 |
| Globus Pallidus (R.GP) | 2.97 | 0.0033 |
| Thalamus (L.Tha) | -2.66 | 0.0085 |
| Thalamus (R.Tha) | -0.59 | 0.5554 |
| Hypothalamus (L.HTh) | -3.52 | 0.0005 |
| Hypothalamus (R.HTh) | -2.42 | 0.0166 |
|  |  |  |
| **Cerebellum** |  |  |
| Cerebellum IX-Tonsil (L.CerIX) | -1.86 | 0.0645 |
| Cerebellum IX-Tonsil (R.CerIX) | -1.54 | 0.1242 |
| Cerebellum VIIIAB-Inferior Posterior -PML (L.CerVIIIAB) | -2.90 | 0.0042 |
| Cerebellum VIIIAB-Inferior Posterior -PML (R.CerVIIIAB) | -2.83 | 0.0051 |
| Cerebellum VIIA-Superior Posterior -Crus I (L.CrusI) | -2.60 | 0.0101 |
| Cerebellum VIIA-Superior Posterior -Crus I (R.CrusI) | -3.20 | 0.0016 |
| Cerebellum VIIA-Superior Posterior -Crus II (L.CrusII) | -3.41 | 0.0008 |
| Cerebellum VIIA-Superior Posterior -Crus II (R.CrusII)* | -3.72 | 0.0003 |
| Cerebellum VI-Superior Posterior (L.CerVI)* | -3.72 | 0.0003 |
| Cerebellum VI-Superior Posterior (R.CerVI)* | -3.62 | 0.0004 |
| Cerebellum V-Anterior B (L.CerVB)* | -3.95 | 0.0001 |
| Cerebellum V-Anterior B (R.CerVB)* | -3.51 | 0.0006 |
| Cerebellum V-Anterior A (L.CerVA)* | -4.30 | 2.7x10^-5^ |
| Cerebellum V-Anterior A (R.CerVA)* | -4.35 | 2.2x10^-5^ |
| Cerebellum IV-Anterior Quadrangulate (L.CerIV)* | -4.23 | 3.7x10^-5^ |
| Cerebellum IV-Anterior Quadrangulate (R.CerIV)* | -4.07 | 6.9x10^-5^ |
| Cerebellum III-Anterior Quadrangulate (L.CerIII) | -3.40 | 0.0008 |
| Cerebellum III-Anterior Quadrangulate (R.CerIII) | -3.19 | 0.0016 |
| Cerebellum II-Anterior Quadrangulate (L.CerII) | -2.90 | 0.0041 |
| Cerebellum II-Anterior Quadrangulate (R.CerII) | -2.76 | 0.0064 |

**Key:** L. refers to region in the left hemisphere, while R. the right hemisphere. * multiple comparisons correction at FWE *p* ≤ 0.05.
