# Supplementary material for: Chimpanzee brain morphometry utilizing standardized MRI preprocessing and macroanatomical annotations
Source: eLife. 2020 Nov 23;9:e60136. doi: 10.7554/eLife.60136 (PMC7723405; doi:10.7554/eLife.60136)
Supplement: Figure 6—source data 1. [file elife-60136-fig6-data1.docx]

**Complete Davi130 labels hemispheric asymmetry**

| **Davi130 Label** | **T-statistic** | **p-value** |
| --- | --- | --- |
| **Leftward Asymmetry** |  |  |
| **Frontal Cortex** |  |  |
| Anterior Superior Frontal Gyrus (aSFG)* | 10.00 | 3.4x10^-19^ |
| Middle Superior Frontal Gyrus (mSFG)* | 5.89 | 1.7x10^-8^ |
| Posterior Superior Frontal Gyrus (pSFG)* | 6.00 | 9.6x10^-9^ |
| Posterior Middle Frontal Gyrus (pMFG)* | 6.94 | 1.7x10^-8^ |
| Lateral Orbitofrontal Cortex (lOFC) | 0.50 | 0.6203 |
|  |  |  |
| **Limbic Cortex** |  |  |
| Entorhinal Cortex (EnC) | 2.36 | 0.0194 |
|  |  |  |
| **Temporal Cortex** |  |  |
| Anterior Insula (aIns)* | 10.64 | 4.2x10^-21^ |
| Posterior Insula (pIns)* | 16.62 | 4.5x10^-39^ |
| Anterior Transverse Temporal Gyrus (aTTG)* | 4.72 | 4.6x10^-6^ |
|  |  |  |
| **Parietal Cortex** |  |  |
| Superior Parietal Lobule (SPL) | 0.31 | 0.7545 |
| Precuneus (PCun)* | 4.00 | 9.2x10^-5^ |
| **Occipital Cortex** |  |  |
| Cuneus (Cun) | 2.25 | 0.0253 |
| **Basal Ganglia** |  |  |
| Nucleus Accumbens (NA)* | 5.21 | 4.9x10^-7^ |
| Basal Forebrain Nuclei (BF)* | 4.02 | 8.5x10^-5^ |
| Globus Pallidus (GP)* | 4.68 | 5.5x10^-6^ |
| Putamen (Pu)* | 8.31 | 1.6x10^-14^ |
|  |  |  |
| **Cerebellum** |  |  |
| Cerebellum IV-Anterior Quadrangulate (CerIV) | 0.58 | 0.5615 |
| Cerebellum III-Anterior Quadrangulate (CerII) | 0.02 | 0.9848 |
|  |  |  |
| **Rightward Asymmetry** |  |  |
| **Frontal Cortex** |  |  |
| Anterior Middle Frontal Gyrus (aMFG)* | -4.30 | 2.8x10^-5^ |
| Anterior Inferior Frontal Gyrus (aIFG)* | -4.57 | 8.7x10^-6^ |
| Middle Inferior Frontal Gyrus (mIFG)* | -6.15 | 4.3x10^-9^ |
| Posterior Inferior Frontal Gyrus (pIFG)* | -6.17 | 3.9x10^-9^ |
| Medial Orbitofrontal Cortex (mOFC)* | -7.05 | 3.1x10^-11^ |
| Frontal Operculum (FOP)* | -17.18 | 9.3x10^-41^ |
| Superior Precentral Gyrus (sPrCG) | -0.59 | 0.5581 |
| Middle Precentral Gyrus (mPrCG)* | -5.56 | 8.9x10^-8^ |
| Inferior Precentral Gyrus (iPrCG) | -2.08 | 0.0386 |
|  |  |  |
| **Limbic Cortex** |  |  |
| Anterior Cingulate Gyrus (ACC)* | -12.50 | 1.2x10^-26^ |
| Middle Cingulate Gyrus (MCC)* | -10.50 | 1.1x10^-20^ |
| Posterior Cingulate Gyrus (PCC)* | -9.38 | 1.8x10^-17^ |
| Parahippocampal Gyrus (PHC) | -0.35 | 0.7287 |
| Amygdala (Amy)* | -5.63 | 6.4x10^-8^ |
| Hippocampus (HC) | -2.94 | 0.0036 |
|  |  |  |
| **Temporal Cortex** |  |  |
| Posterior Transverse Temporal Gyrus (pTTG)* | -3.93 | 0.0001 |
| Anterior Superior Temporal Gyrus (aSTG)* | -3.53 | 0.0005 |
| Posterior Superior Temporal Gyrus (pSTG)* | -9.94 | 6.5x10^-19^ |
| Anterior Middle Temporal Gyrus (aMTG)* | -4.54 | 9.9x10^-6^ |
| Posterior Middle Temporal Gyrus (pMTG)* | -3.61 | 0.0004 |
| Anterior Inferior Temporal Gyrus (aITG) | -0.52 | 0.6044 |
| Posterior Inferior Temporal Gyrus (pITG) | -2.97 | 0.0034 |
| Anterior Fusiform Gyrus (aFFG)* | -3.62 | 0.0004 |
| Posterior Fusiform Gyrus (pFFG)* | -9.48 | 9.4x10^-18^ |
| **Parietal Cortex** |  |  |
| Superior Postcentral Gyrus (sPoCG) | -2.37 | 0.0187 |
| Middle Postcentral Gyrus (mPoCG)* | -7.91 | 2.0x10^-13^ |
| Inferior Postcentral Gyrus (iPoCG)* | -5.50 | 1.1x10^-7^ |
| Paracentral Lobule (PCL) | -0.45 | 0.6517 |
| Angular Gyrus (AnG)* | -7.55 | 1.7x10^-12^ |
| Parietal Operculum (POP)* | -9.85 | 8.4x10^-19^ |
| Supramarginal Gyrus (SMG)* | -4.41 | 1.7x10^-5^ |
|  |  |  |
| **Occipital Cortex** |  |  |
| Lingual Gyrus (LG) | -1.76 | 0.0801 |
| Calcarine Sulcus (Calc)* | -6.78 | 1.4x10^-10^ |
| Superior Occipital Gyrus (sOG) | -2.34 | 0.0204 |
| Middle Occipital Gyrus (mOG)* | -10.84 | 1.1x10^-21^ |
| Inferior Occipital Gyrus (iOG) | -1.98 | 0.0491 |
|  |  |  |
| **Basal Ganglia** |  |  |
| Caudate Nucleus (CN)* | -14.53 | 8.4x10^-33^ |
| Thalamus (Th)* | -15.86 | 8.4x10^-37^ |
| Hypothalamus (HTh) | -1.45 | 0.1486 |
| **Cerebellum** |  |  |
| Cerebellum IX (CerIX)* | -5.67 | 5.1x10^-8^ |
| Cerebellum VIIIAB (CerVIII)* | -3.82 | 0.0002 |
| Cerebellum VIIA – Crus I (CrusI)* | -3.38 | 0.0009 |
| Cerebellum VIIA – Crus II (CrusII)* | -10.36 | 2.8x10^-20^ |
| Cerebellum VI (CerVI)* | -4.73 | 4.3x10^-6^ |
| Cerebellum V-Anterior B (CerVB) | -1.44 | 0.1522 |
| Cerebellum V-Anterior A (CerVA) | -3.07 | 0.0024 |
| Cerebellum III (CerIII) | -0.05 | 0.9607 |
|  |  |  |

**Key:** * multiple comparisons correction at FWE *p* ≤ 0.05.
